# Live cell immunogold labelling of RNA polymerase II

**DOI:** 10.1038/srep08324

**Published:** 2015-02-09

**Authors:** Igor Orlov, Andreas Schertel, Guy Zuber, Bruno Klaholz, Robert Drillien, Etienne Weiss, Patrick Schultz, Danièle Spehner

**Affiliations:** 1 Department of Integrated Structural Biology, IGBMC (Institut de Génétique et de Biologie Moléculaire et Cellulaire) INSERM, U964; CNRS/Strasbourg University, UMR7104 1, rue Laurent Fries, BP10142 67404 Illkirch, France; 2 Carl Zeiss Microscopy GmbH, Training, Application and Support Center (TASC), Carl-Zeiss-Straße 22, D-73447 Oberkochen, Germany; 3 LCAMB, UMR 7199, CNRS/Strasbourg University, Faculty of Pharmacy, 74 route du Rhin, 67401 Illkirch, France; 4 LBSC, UMR 7242, Institut de Recherche de l'Ecole de Biotechnologie de Strasbourg, Strasbourg University, 300 Boulevard Sébastien Brandt, BP 10413, 67412 Illkirch, France

## Abstract

Labeling nuclear proteins with electron dense probes in living cells has been a major challenge due to their inability to penetrate into nuclei. We developed a lipid-based approach for delivering antibodies coupled to 0.8 nm ultrasmall gold particles into the nucleus to label RNA polymerase II. Focussed Ion Beam slicing coupled to Scanning Electron Microscopy (FIB/SEM) enabled visualization of entire cells with probe localization accuracy in the 10 nm range.

The intracellular localization and dynamics of proteins involved in cellular processes are most often studied in living cells at light microscopy resolution (~300–500 nm) by monitoring proteins fused to fluorescent tags. The limited spatial definition and the fluorescence-restricted information content of light microscopy can be complemented by electron microscopy (EM) which expands resolution to molecular dimensions and uncovers the ultracellular context of the protein of interest. Macromolecular detection in EM relies on labeling with electron-dense particles and the most successful method conjugates primary or secondary antibodies to gold particles that provide high contrast and easily recognizable shape^1^. In most experiments the labeling step is performed after specimen embedding and the antibodies are incubated either with fixed resin-embedded cell sections^2^ or sucrose embedded cryosections^3^. Poor epitope accessibility within cell sections and the requirement of fixatives leading to denaturation, even when reduced in the case of sucrose embedding, restrict detection of rare epitopes. Pre-embedding methods were developed to label the macromolecules before processing the cells for EM^4^,^5^ but such *in situ* immunolabelling experiments required chemical fixation and cell permeabilization, which affect the structural integrity of the sample. Furthermore the gold-conjugated antibodies failed to cross the nuclear envelop and reach their targets in the cell nucleus.

Here we have developed a method to internalize immunogold labels into living cells and allow their nuclear import. Physiological internalization is generally restricted to specialized cell types involved in macropinocytosis or receptor mediated endocytosis^6^. For more general use we employed lipid-based protein delivery agents compatible with cell viability to internalize the probes^7^. The cationic lipid dioctadecylamidoglycine spermine (DOGS) was first described as an effective gene transfection reagent^8^ and such lipids were later used to deliver antibodies into the cytoplasm of mammalian cells^9^,^10^. Assays with E6 oncoprotein neutralizing antibodies have demonstrated that the lipid vehicles protect the antibodies from degradation in the endosomal/lysosomal compartment since they inhibit E6 activity *in vivo*^11^. Antibodies do not diffuse freely into the nucleus and fluorescein-labelled anti tubulin antibodies delivered by lipid vehicles into HeLa cells remain cytoplasmic as expected for molecules devoid of Nuclear Localization Signals^12^ (Fig. S1). However antibodies directed against nuclear targets such as RNA polymerase II (RNAPII) can be imported into the nucleus presumably because they recognize their target in the cytoplasm and are transported into the nucleus by a piggyback mechanism.

## Results

**Figure 1:**
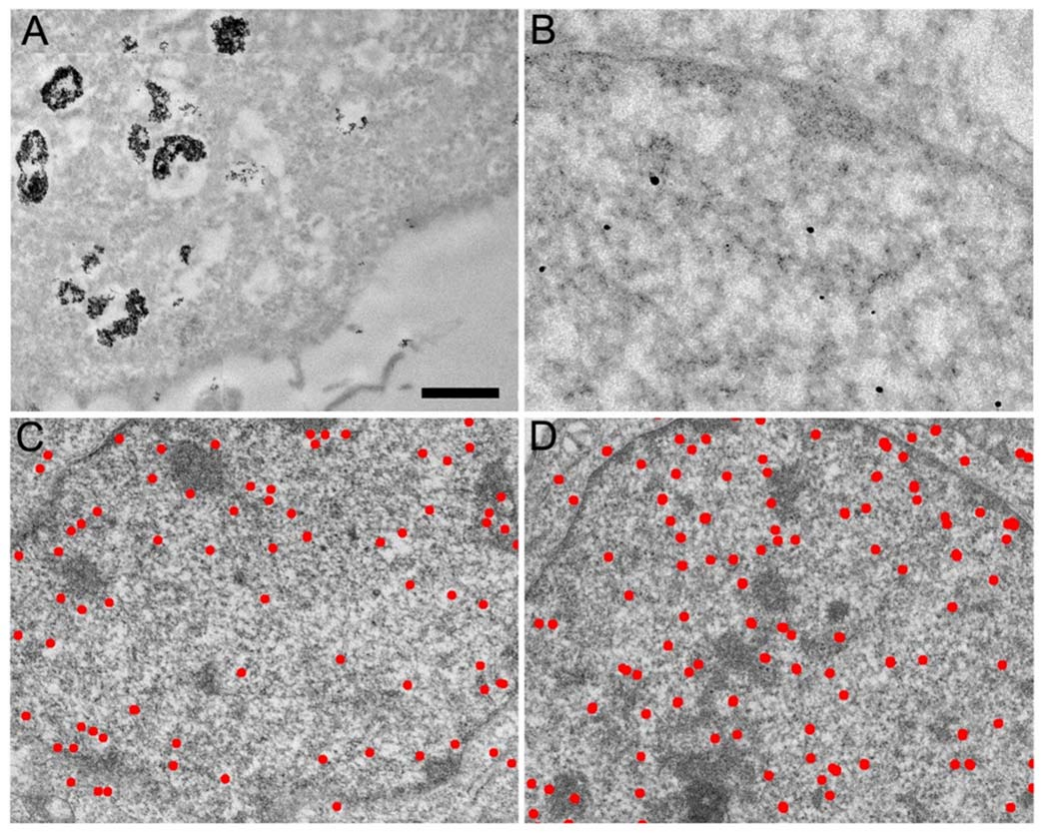
Transmission electron micrographs of HeLa cell sections labeled *in vivo* with antibodies directed against RNA polymerase II and coupled to gold beads. (A) Antibodies coupled to 6 nm colloidal gold particles are found aggregated in large cytoplasmic vesicles. (B) Antibodies coupled to 0.8 nm gold particles are detected as individual spots after silver enhancement in the cytoplasm and are enriched in the nucleus. (C) Distribution of the amplified gold particles coupled to IgG molecules directed against RNA polymerase II. (D) Distribution of the amplified gold particles coupled to Fab fragments directed against RNA polymerase II. The bar represents 1 μm in (A), 0.2 μm in (B) and 0.6 μm in (C) and (D).

To label RNAPII with electron dense probes suitable for EM localization studies we employed a monoclonal antibody directed against the C-terminal domain (CTD) of its largest subunit (αRNAPII, clone 7C2). This antibody was characterized previously for its ability to label RNAPII *in vivo* in cultured HeLa cells^12^. It was here coupled to 6 nm colloidal gold particles (αRNAPII-gold6) and introduced into living HeLa cells using a commercially available lipid-based antibody delivery system. After 8 h incubation, the αRNAIPII-gold6 was mostly located in big patches within cytoplasmic vesicles and at the periphery of the cell membrane (Fig. 1A). Control cells without antibody delivery did not uptake the αRNAPII-gold6 particles. In sharp contrast to the fluorescent probe, αRNAPII-gold6 could not be detected in the nucleus indicating that the internalized probe could not cross the nuclear envelope.

To determine whether the size of the colloidal gold particles could affect nuclear transport, the αRNAPII IgG was conjugated to 0.8 nm ultrasmall colloidal gold (αRNAPII-US)^13^. The reduced size of the gold particle will affect mass, charge and volume of the final marker since several 15 nm sized antibodies can bind to a single 6 nm gold particle while it is generally believed that one or two US gold particles can bind to each IgG molecule. The freshly prepared αRNAPII-US was delivered into HeLa cells as described above and after 8 h incubation the cells were processed for Transmission Electron Microscopy (TEM) observation. Due to their small size, the US gold particles were amplified to be visible at the TEM level. The observation of 100 nm thick cell sections readily disclosed the amplified gold particles which were present in the cytoplasm and enriched in the nucleus (Fig. 1B). The cytoplasmic αRNAPII-US particles were not found in vesicles indicating that they are released more efficiently into the cytosol than αRNAPII-gold6. Most importantly, the US-coupled αRNAPII was efficiently imported into the nucleus where it accumulated similarly to the control fluorescent antibody^12^. We further reduced the size of the probe by conjugating the US gold to Fab fragments (αRNAPII-Fab-US). When used in the same conditions as the αRNAPII-USG, the smaller αRNAPII-Fab-US showed an improved cellular uptake and was similarly enriched in the nucleus (Fig. 1C and D, Fig. S2). Additional experiments were performed by conjugating αRNAPII to a solution of gold particles with a size ranging between 2 and 5 nm. As for the 6 nm gold particles, these experiments revealed most of the gold conjugates as aggregates in lipids vesicles (Fig. S3). Altogether these observations indicate that antibodies or Fab fragments conjugated to 0.8 nm gold particles are efficiently delivered into living cells, can be imported into the nucleus and detected by TEM after amplification.

**Figure 2:**
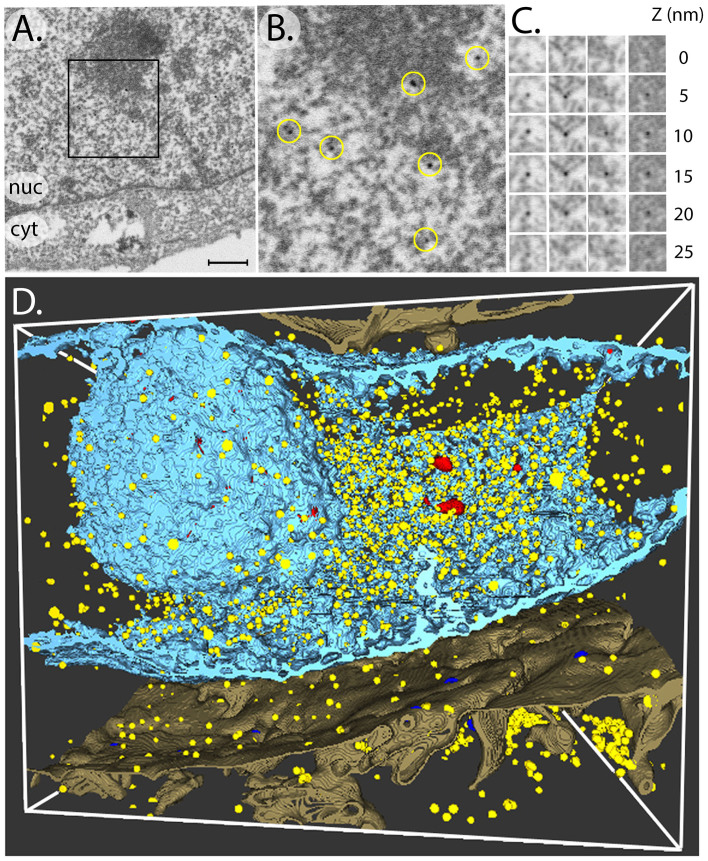
FIB/SEM visualization of amplified gold particles. (A) Scanning Electron Micrograph of a HeLa cell labeled *in vivo* with RNA polymerase II-specific Fab fragments conjugated to ultrasmall gold particles. The nuclear (nuc) and cytoplasmic (cyt) compartments are indicated. The bar represents 0.5 μm. (B) Enlargement of the area delineated in (A) to show the amplified gold particles highlighted arrowheads circles. (C) Gallery of 4 amplified gold particles (columns) imaged by SEM on successive 5 nm thick FIB sections (D) Three-dimensional reconstruction of a series of FIB/SEM images representing the whole nucleus. The electron dense nuclear envelope and the nucleolus are represented by blue isodensity surfaces. The amplified gold particles are represented in yellow. Dense heterochromatin domains are represented in red.

The FIB/SEM technology was used to map the distribution of RNAPII in the entire nuclear volume. In this method, the focused ion beam (FIB) mills away a thin section of the samples while the scanning electron beam (SEM) is used to image the newly created cell cross-section^14^. The sequential use and iteration of these two processes opens the possibility to image large areas at high spatial resolution and over a depth of several microns. To date FIB/SEM imaging has not been coupled with *in situ* live cell labeling and little is known about the visualization of gold particles^15^. The combination of these two methods offers the unique possibility to quantify the labeled nuclear proteins within the whole nucleus. SEM images 30 by 20 μm in size of HeLa cells labeled with αRNAPII-US or αRNAPII-Fab-US were recorded with an image pixel size of 5 nm. Sequential FIB milling removed 25 nm thick slices and a stack of 400 images was recorded to yield a volume 10 μm in depth. The amplified gold particles were readily detected in the SEM images (Fig. 2A–B). The high contrast obtained possibly reflects the absence of superposition effects with other cellular structures. At the low SEM acceleration potential used the collected back scattered electrons have a penetration depth of 5–10 nm for osmium treated cells as modeled by Monte Carlo simulations^16^,^17^ and therefore only structures close to the FIB-milled surface are imaged.

In order to quantify the labeling it is crucial to know which proportion of gold particles is detected within the milled-away 25 nm thick slices. Depending on their depth with respect to the imaged surface the bead size and contrast may be variable with the risk for some particles to be missed. To address this question, the thickness of the FIB slices was reduced to 5 nm in order to trace the same particle on successive views (Fig. 2C). In these conditions the same particle was detected on average on 4 consecutive sections consistent with its size of about 20 nm. We therefore expected to miss about 20% of the particles by milling 25 nm slices. Moreover, the milling of 5 nm thick slices positions the gold particle with high precision since its central coordinates can be calculated by interpolation at sub-pixel resolution in x, y and z directions. A total of 100 successive sections (roughly 2.5 μm) were analyzed for the αRNAPII-US and the αRNAPII-Fab-US markers. The amplified gold particles were counted, the labeling density was determined in different cellular compartments (Table 1), and the particle distribution was represented in the 3-D cellular context (Fig. 2D, Fig. S4 and Movie1). The uptake efficiency as monitored by the average cellular density of particles was found to be two fold higher for the Fab-US (16.8 particles/μm^3^) than for the IgG-US (4.9 particles/μm^3^) indicating again that smaller markers enter better into the cell. Consistently with the behavior of the fluorescently labeled αRNAPII IgG and the TEM observations, 84% and 79% of the amplified gold particles were found in the nucleus; and the labeling density was 5.0 times and 7.9 times higher in the nucleus than in the cytoplasm for the Fab-US and for the IgG-US, respectively. This indicates a strong enrichment of the gold particles in the nucleus and an active nuclear translocation of the conjugated Fab or IgG molecules. The αRNAPII-Fab-US and αRNAPII-IgG-US particles were mostly localized in the euchromatin region of the nucleus which is consistent with the distribution of active transcription in the less densely packed chromatin regions of the nucleus. Moreover the nucleolus showed a 3.2 fold lower αRNAPII-Fab-US labeling density which is expected since no RNAPII transcription occurs in this nuclear compartment. This depletion was less pronounced in the case of the IgG-based marker.

## Discussion

Transcription is believed to occur in specialized foci locally enriched in RNAPII molecules^18^ and recent data suggest that this dynamic clustering is regulated by external cellular signals^19^. The RNAPII molecules were however found distributed in the whole nucleus. Our data is consistent with an overall distribution of RNAPII in the whole nuclear volume but does not reveal significant clustering of the enzymes since the vast majority of the gold particles are separated by more than 200 nm. Occasionally two gold particles can be found in close proximity and we cannot exclude that this corresponds to two antibodies bound to the same RNA polymerase since the 7C2 antibody recognizes both hyperphosphorylated and unphosphorylated forms of the 52 heptapeptide repeats found in the CTD. In order to estimate the fraction of labelled RNAPII molecules we multiplied the labelling density obtained for the best probe (23.8 αRNAPII-Fab-US particles per μm^3^, Table 1) by the volume of the HeLa cell nucleus (with a diameter of ~14 μm the nucleus occupies a volume of about 1400 μm^3^). A total of 33.000 RNAPII molecules are therefore labeled in our conditions, which corresponds to about 10% of the estimated enzymes per cell nuclei^17^. With the currently achieved labeling efficiency it is therefore unlikely to reveal clustered RNAPII molecules. Post-embedding immunogold labeling experiments using the 7C2 antibody were previously performed on Lowicryl sections of HeLa cells infected by type 1 herpes simplex virus and revealed that RNAPII were associated with viral gene transcription factories and clusters of interchromatin granules^20^. Discrete nuclear structures such as perichromatin fibrils or interchromatin granules previously identified as sites of RNA transcription and maturation^21^,^22^ were not specifically labeled with our pre-embedding immunogold labeling method. Additional experiments are needed to assess whether the hypophosphorylated RNAPII labeled in the cytoplasm can still be recruited to gene promoters and phosphorylated to be converted into a transcriptionally active state.

In summary we demonstrate that lipid-based delivery systems allow the efficient internalization of electron dense markers coupled to antibodies. Their fate depends on the size of the gold particle and only antibodies coupled to 0.8 nm gold particles are delivered into the cytoplasm and are not sequestered in membrane structures. The FIB/SEM visualization allows a clear detection and quantification of the amplified gold particles in the whole nucleus. The probes and the delivery system are compatible with cell viability and the localization of the RNAPII as revealed by the markers is consistent with the expected distribution of the enzyme. This new method holds promises for high resolution in situ labeling of cellular proteins and for cryo electron tomography.

## Methods

### Antibody probes

We used a monoclonal antibody directed against the C-terminal domain of the largest subunit of RNA polymerase II (clone 7C2)^23^. The immunopurified antibody was either fluorescently labeled using fluorescein isothiocyanate (FITC) using standard methods or coupled to 6 nm gold colloid particles^1^. To generate Fab fragments 1 mg of immunopurified 7C2 antibody was cleaved into Fab fragments by immobilized Ficin and purified using a protein A column (Pierce Biotechnology). The IgG and the Fab were coupled to US-colloïdal gold particles by the manufacturer (US-colloidal gold, Aurion, The Netherlands).

### Antibody delivery

Ten μl of purified 7C2 antibodies or of the different 7C2 gold conjugates at a protein concentration of 100 μg/ml were incubated for 10 min at room temperature with 3 μl of a lipid based formulation allowing the delivery of functional antibodies into living cells (Ab-DeliverIN™, Ozbiosciences). The volume was completed to 300 μl. 200 μl of the formed lipoplexes were added into the culture media of adherent HeLa cells which were 50–70% confluent at the time of the delivery. The cells were incubated for 4, 8, 12 or 24 hours with the protein delivery agent and subsequently washed with PBS before being fixed with 2.5% glutaraldehyde diluted in PBS for at least 1 hour. After several washes with water, the silver enhancement was done according to the manufacturer's protocol (SE-EM, Aurion, The Netherlands) and washed again with water.

### Specimen preparation and Transmission Electron Microscopy

The glutaraldehyde crosslinked cells were processed for electron microscopy using standard protocols. Briefly, cells were fixed and contrasted with 0.5% osmium for 20 min then contrasted with 2% uranyl acetate before being dehydrated in increasing concentrations of alcohol and flat embedded in epon. The resin embedded cells were sectioned into 100 nm thick slices that were deposited on an electron microscopy grid and observed in a Transmission Electron Microscope (Tecnai F20 G2, FEI) equipped with a field emission gun operating at 200 kV at a magnification of 11.500 x, on a 2048 × 2048 CCD camera (Ultrascan 1000, Gatan Inc., Pleasanton) with a final pixel spacing on the specimen of 0.92 nm. Adjacent regions of the specimen were recorded and the images were stitched together to form montages of 5 × 5 frames by using the Serial EM software^24^.

### Scanning Electron microscopy and Focused Ion Beam milling

Focused Ion Beam milling and Scanning Electron Microscopy observations were performed using a dedicated instrument (Auriga 60, Carl Zeiss Microscopy GmbH, Oberkochen). For 3D reconstruction 25 nm thick slices of the resin embedded sample were removed by FIB milling and the freshly exposed cross-section was imaged with a lateral pixel size of 5 nm in a serial manner. For FIB milling the probe current was set to 2 nA at 30 kV acceleration potential. The FIB-milled cross-section width was of 30 μm by 20 μm and the milling depth was 10 μm. The image store resolution was 2048 pixel × 1536 pixel resulting in an image width of about 10 μm and in an image height of about 7.5 μm. For noise reduction line averaging with line averaging count number N = 11 and scan speed 4 was used. The resin block was glued on an aluminum stub using silver paint (Silver dag 1415, Plano GmbH, Wetzlar). First all sidewalls besides the block face were covered by silver paint and then the block face was sputter-coated with a few nanometer thick metal film to avoid any charging of the resin. Prior to serial cross-sectioning and imaging no protection layer besides the sputter coating was deposited. The SEM acceleration voltage was set to 1.5 kV, the SEM aperture was 60 μm and high current mode was turned on. For imaging the Energy selective Back-scattered electron (EsB) detector was used with a retarding EsB grid voltage of 1425 volts. The SEM imaging took about 29 sec and the milling of each slice about 4 sec. The acquired data cube consists of more than 400 single slice images. The grey level scale was inverted in order to obtain a TEM like image.

### Image processing and segmentation

A stack containing 300 images 2048 by 1536 pixels in size was created after inverting and normalizing the image intensities. A cropped region 1024 by 1024 pixels in size from the center of the stack was aligned using the “multistack Reg Fix” plugin in Fiji^25^. Segmentation and 3-D rendering of the density map were performed using the Seg2D software (http://www.seg3d.org). The segmentation was done in a semi-automatic mode by thresholding the density map and tracing continuous grey levels. Particle detection was done by setting a density threshold based on the high contrast of the amplified gold particles, while particle counting was performed in Fiji.

## Author Contributions

I.O. contributed to data acquisition and analyzed the tomogram data with BK, A.S. acquired the FIB/SEM data, G.Z. conjugated antibodies, R.D. cultured cells and internalized the labels, E.W. controlled internalization with fluorescent labels, D.S. designed and prepared the samples for electron microscopy, P.S. and D.S. wrote the manuscript. All authors reviewed the manuscript.

## Supplementary Material

Supplementary Information Supplemental information

Supplementary Information supplemental movie

## Figures and Tables

**Table 1:** Quantification of the gold particles in different cellular compartments

	αRNAPII-Fab-US			αRNAPII-IgG-US		
	number	volume (μm^3^)	density	number	volume μm^3^	density
Cytoplasm	196	41.9	4.7	124	101.9	1.2
Whole nucleus	1717	71.9	23.8	804	86.8	9.3
Nucleus without nucleoli	1699	69.5	24.4	757	80.1	9.5
Nucleoli	18	2.4	7.5	47	6.7	7.0
total	1913	113.8	16.8	928	188.7	4.9
