# Supplementary material for: Live cell immunogold labelling of RNA polymerase II
Source: Sci Rep. 2015 Feb 9;5:8324. doi: 10.1038/srep08324 (PMC4321181; doi:10.1038/srep08324)
Supplement: Supplementary Information — Supplemental information [file srep08324-s1.pdf]

SUPPLEMENTAL MATERIAL

LIVE CELL IMMUNOGOLD LABELLING OF RNA POLYMERASE II

Igor Orlov, Andreas Schertel, Guy Zuber, Robert Drillien, Etienne Weiss, Patrick Schultz, Danièle Spehner

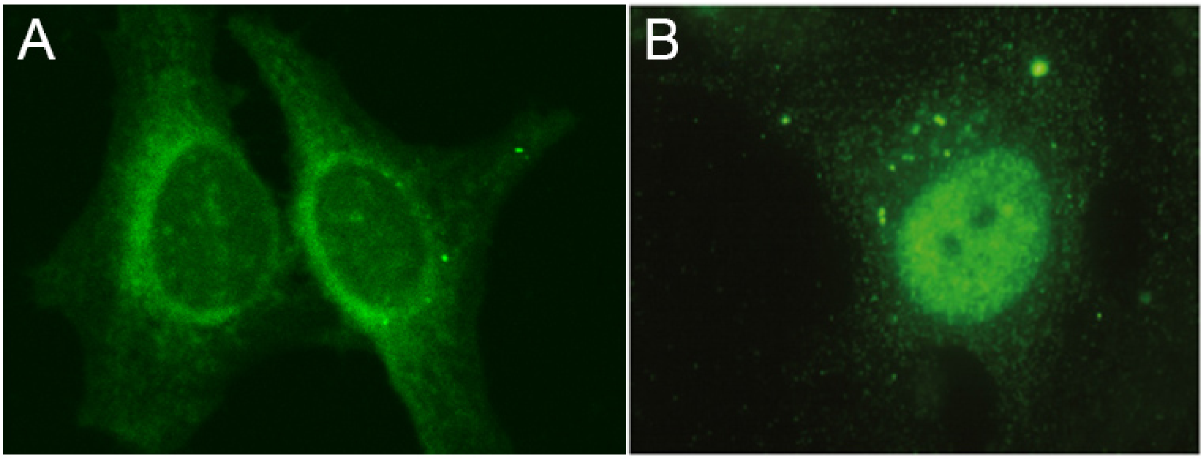

Supplemental figure 1 Live cell fluorescent imaging. (A) Live cell imaging of fluorescein-labeled anti-tubulin IgGs after delivery with DOGS showing that Abs diffuse in the cytosol but do not enter the nucleus. (B). Immunodetection of anti-RNA Pol II IgGs (7C2) after lipid mediated delivery showing that in this case Abs are cargoed into the nucleus but do not diffuse into the RNA pol II depleted nucleolus.

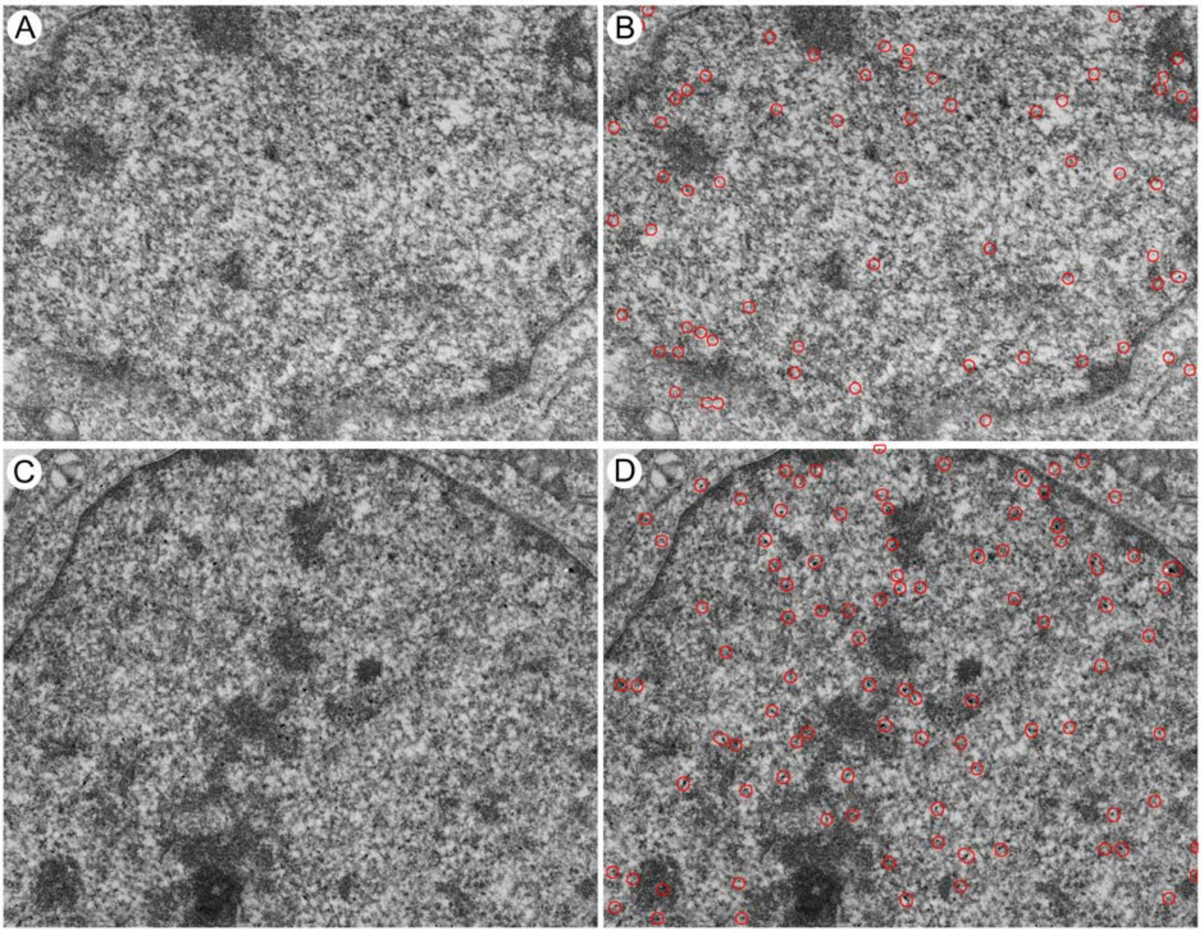

Supplemental figure 2: Full resolution transmission electron micrographs of HeLa cell sections labeled *in vivo* with 7C2 antibodies directed against RNA polymerase II. The antibodies were conjugated to 0.8 nm gold beads and revealed by silver enhancement. (A) In vivo labelling with full IgG molecules conjugated to gold particles (B) The amplified gold bead were highlighted with circles (C) In vivo labelling with Fab fragments conjugated to gold particles (D) The amplified gold bead were highlighted with circles.

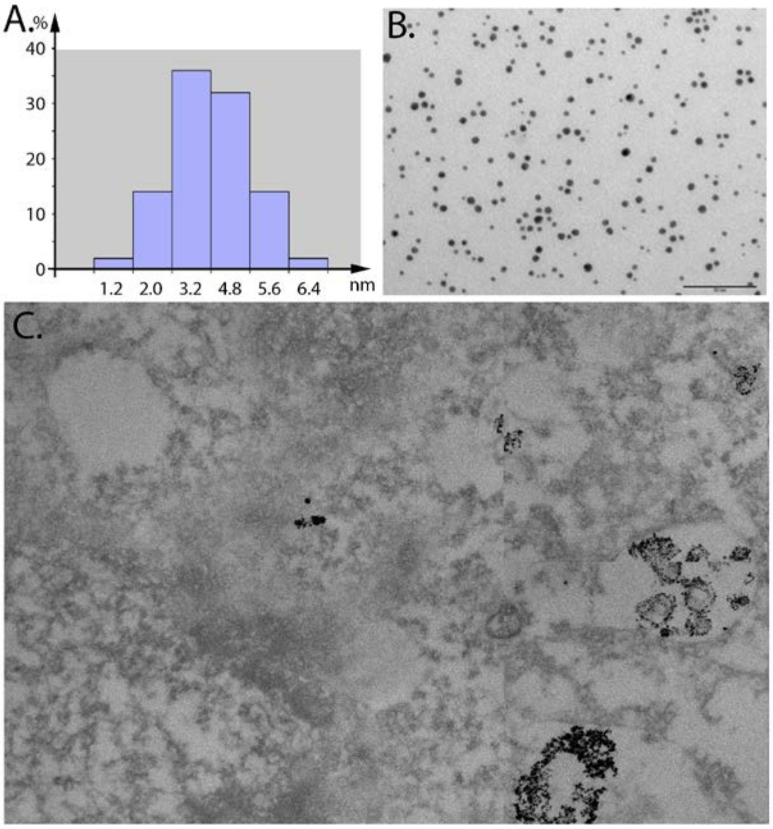

Supplemental figure 3: HeLa cell sections labeled *in vivo* with 7C2 antibodies coupled to gold particles with a size ranging from 2 to 6 nm. (A) Size distribution of the gold particles. (B) Transmission electron micrograph of isolated gold particles used in the labeling experiment. (C) Transmission electron micrographs of HeLa cell sections labeled *in vivo* with 7C2 antibodies coupled to gold particles with a size ranging from 2 to 6 nm.

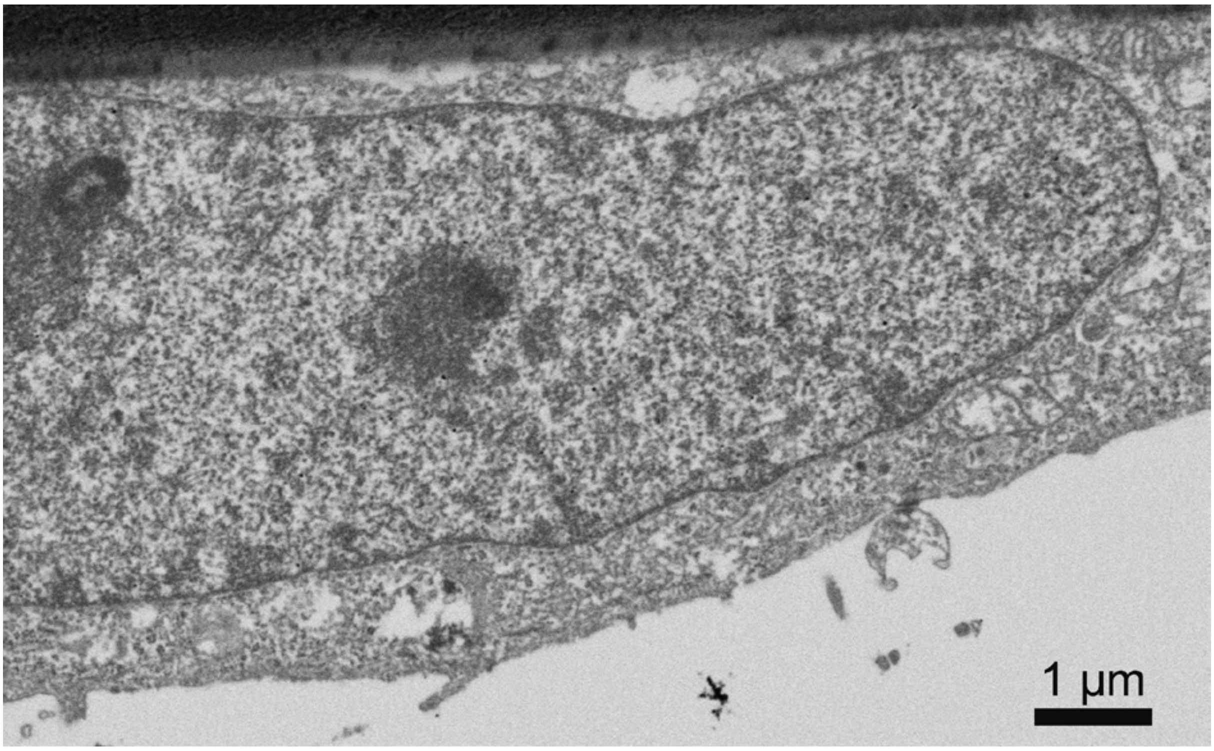

Supplemental figure 4: Scanning Electron Microscopy of a HeLa cell labeled *in vivo* with 7C2 Fab fragments coupled to 0.8 gold particles. Overview showing the area of the cell analyzed for the quantification of gold particles binding to the different cell compartments and reported in Table 1. This image corresponds approximately to the middle of the image stack created by successive milling and imaging with the FIB/SEM. The top of the image correspond to the support on which the cell was growing while the bottom corresponds to the media from which the lipoplexes were delivered.

Supplemental movie 1: Whole stack of images of a HeLa cell labeled *in vivo* with 7C2 Fab fragments coupled to 0.8 gold particles. This stack of successive images corresponds to the area of the cell analyzed for the quantification of gold particles binding to the different cell compartments and reported in Table 1.
